# (William) Ritchie Russell (1903–1980)

**DOI:** 10.1007/s00415-024-12325-0

**Published:** 2024-03-26

**Authors:** Jonathan E. Attwood, Juliette Houchois, Margaret M. Esiri, Edward H. F. de Haan, Gabriele C. DeLuca

**Affiliations:** 1 Nuffield Department of Clinical Neurosciences, University of Oxford, Oxford, UK; 2 St Hugh’s College, Oxford, UK; 3 Donders Institute, Radboud University, Nijmegen, The Netherlands

**Fig. 1:**
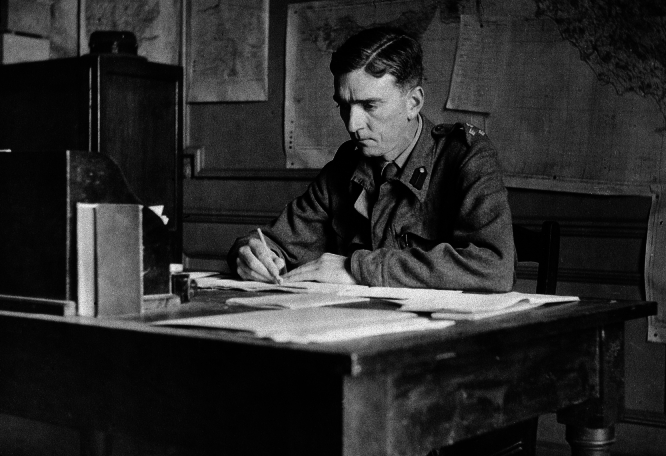
Ritchie Russell photographed by Brigadier Sidney Smith (Wellcome Collection, via Wikimedia Commons)

In the aftermath of World War II (WWII), a young neurologist was sat at a desk in a corridor, which he called his office, organising index cards, which his colleagues affectionately referred to as his “folly” [1] (Fig. 1). This man was Ritchie Russell, who, if he is known at all today, is probably remembered for establishing the link between post-traumatic amnesia (PTA) and severity of traumatic brain injury (TBI). Russell would go on to become the first professor of clinical neurology at the University of Oxford, where his legacy can still be felt today. But the story of how he became a pioneer in neurology holds valuable lessons for everyone dedicated to enhancing the lives of people with neurological conditions.

William Ritchie Russell was born in 1903, the same year which saw the formation of the suffragette movement. Ten years earlier, his mother Beatrice Ritchie (whose maiden name would become his preferred name), gained a medical degree from the University of Brussels, having been one of the first women to study medicine in Scotland [2]. Although fully qualified, she was denied a license to practice medicine. When war broke out in 1914, women were still not allowed to join the Royal Army Medical Corps. Instead, Beatrice joined the Scottish Women’s Hospitals movement, through which more than a thousand women volunteered to run a network of fourteen hospitals across Europe. In Edinburgh, Beatrice led the personnel committee, orchestrating the people and resources that sustained this network, working ‘quietly and unostentatiously from beginning to end’ [2]. Her spirit and character are vividly reflected in the remarkable contributions her son made when conflict returned to Europe during WWII.

Ritchie Russell studied medicine at the University of Edinburgh, where his father was professor of medicine. He trained at the Edinburgh Royal Infirmary and spent two years at the National Hospital in Queen Square, London. In 1940, as the crisis of WWII deepened, a national Military Hospital for Head Injuries (MHHI) was established in Oxford by the neurosurgeon Hugh Cairns [3]. Following the publication of his MD thesis, in which he established the link between PTA and TBI, Ritchie Russell was appointed to the neurology unit at the MHHI by Charles Symonds [3, 4]. Suddenly, Russell found himself at the center of a trauma network stretching across the Western Front. Mobile neurosurgical units stabilized soldiers with head injuries who were then transported back to Oxford for further treatment. This unique setting became a hub for medical innovation, pioneering the use of antibiotics, refining modern neurosurgical techniques, and leading to the inception of the paralympic games, among many notable achievements [5].

From Ritchie Russell’s perspective, admission to the MHHI marked the start of a life-long journey of rehabilitation and opportunities to participate in research for each patient. The care and study of these veterans became his life’s work. Russell was a pioneer in recognizing the power of medically supervised rehabilitation to support people living with neurological conditions. He was among the first to incorporate physical and occupational therapy as active components of treatment. He also grasped the value of care being coordinated by a neurologist whose relationship with each patient began at their initial assessment and who followed their progress closely for years. What’s more, he appreciated that patients who had the opportunity to participate in long-term multi-disciplinary care would be highly motivated to engage with longitudinal research that could yield valuable insights into their condition.

Coordinating such comprehensive care and assessment in an era preceding both England’s National Health Service (NHS) and personal computing was no mean feat. Russell achieved this by maintaining contact with thousands of patients and forming a centralized repository of searchable data, adopting an unglamorous organizational role in a manner reminiscent of his mother in the Scottish Women’s Hospitals. After the war, he embarked on an ambitious project combining postal surveys with a novel system to systematically store clinical data in a binary format using edge-punched index cards. This system enabled him to identify groups of patients with specific characteristics by threading a needle through holes punched along the edge of stacked cards. Using this method, he reported the outcomes of 1,166 patients over five years, establishing the burden of post-traumatic epilepsy, disability, and unemployment among survivors of penetrating brain injuries for the time [6]. Not bad for a “folly”!

By the end of the Korean War in 1953, Russell had defined a cohort of more than 3,000 veterans living with the effects of brain injuries ranging from concussions to gunshot wounds. Even now, the MHHI cohort remains one of the largest datasets describing long-term outcomes after TBI ever assembled, comparable only with the study of veterans who returned from the Vietnam War. Russell continued to monitor the progress of these men for decades, inviting many back to Oxford for further assessment and synthesizing his observations in influential monographs on traumatic aphasia (1961) and traumatic amnesia (1971) [7, 8].

In 1963, Russell handed the ongoing care of the MHHI veterans over to Dr Freda Newcombe, who continued his work combining neuropsychological assessments with lesion localization to produce seminal insights into mechanisms of language, memory, and emotion [9]. Around the turn of the twenty-first century, as many members of the cohort reached the end of their lives, a small number donated their brains to the Oxford Brain Bank. In this way, Russell’s vision resulted in a truly unique combination of clinical, neuropsychological, neuroradiological, and neuropathological data spanning more than 70 years. Today, these resources are still being studied to further our understanding of the long-term effects of TBI and its links with neurodegenerative diseases.

Russell’s contributions extended far beyond his work at the MHHI, from combatting the *poliomyelitis* epidemic to the design of retirement flats in his final years [10]. His story is testament to the virtues of unglamorous work, the value of long-term investment in science, and, above all, how combining comprehensive care with longitudinal research can benefit the patients of today and tomorrow.
